# Non-Intrusive Monitoring and Detection of Mobility Loss in Older Adults Using Binary Sensors

**DOI:** 10.3390/s25092755

**Published:** 2025-04-26

**Authors:** Ioan Susnea, Emilia Pecheanu, Adina Cocu, Adrian Istrate, Catalin Anghel, Paul Iacobescu

**Affiliations:** Department of Computers and Information Technology, University Dunarea de Jos of Galati, 800008 Galati, Romania; adina.cocu@ugal.ro (A.C.); adrian.istrate@ugal.ro (A.I.); catalin.anghel@ugal.ro (C.A.); paul.iacobescu@ugal.ro (P.I.)

**Keywords:** mobility loss, binary sensors, anomaly detection, time series forecasting, behaviorally meaningful places

## Abstract

(1) Background and objective: Mobility is crucial for healthy aging, and its loss significantly impacts the quality of life, healthcare costs, and mortality among older adults. Clinical mobility assessment methods, though precise, are resource-intensive and economically impractical, and most of the existing solutions for automatic detection of mobility anomalies are either obtrusive or improper for long time monitoring. This study explores the feasibility of using non-intrusive, low-cost binary sensors for continuous, remote detection of mobility anomalies in older adults, aiming to identify both sudden mobility events and gradual mobility loss. (2) Method: The study utilized publicly available datasets (CASAS Aruba and HH120) containing annotated activity data recorded from binary sensors installed in residential environments. After data preprocessing—including filtering irrelevant sensor events and aggregation into behaviorally meaningful places (BMPs)—a time series forecasting model (Prophet) was used to predict normal mobility patterns. A fuzzy inference module analyzed deviations between observed and predicted sensor data to determine the probability of mobility anomalies. (3) Results: The system effectively identified periods of prolonged inactivity indicative of potential falls or other mobility disruptions. Preliminary evaluation indicated a detection rate of approximately 77–81% for point mobility anomalies, with a false positive rate ranging from 12 to 16%. Additionally, the approach successfully detected simulated gradual declines in mobility (1% per day reduction), evidenced by statistically significant regression trends in activity levels over time. (4) Conclusions: The study argues that non-intrusive binary sensors, combined with lightweight forecasting models and fuzzy inference, may provide a practical and scalable solution for detecting mobility anomalies in older adults. Although performance can be further enhanced through improved data preprocessing, predictive modeling, and anomaly threshold tuning, the proposed system effectively addresses key limitations of existing mobility assessment approaches.

## 1. Introduction

### 1.1. Background of This Study

Broadly defined as “one’s ability to move independently around their environment” [1], mobility is crucial for healthy aging and for the wellbeing of the elderly [2]. Limited mobility is an important predictor of hospitalization incidence, disability, and mortality, leading to a significant increase in medical expenses and a decrease in quality of life [3].

Limitations in mobility increase with age, affecting 35% of people aged 70 and the majority of those over 85 [4].

Mobility is influenced by a range of interconnected factors. As people age, their physical abilities gradually decline. Changes in gait, balance, strength, and overall muscle function—often associated with conditions like sarcopenia—can significantly impact mobility. Beyond these physical changes, neuromuscular alterations also contribute to mobility decline. The loss of motor units, a reduction in muscle fiber size, and disruptions in nerve–muscle communication all make movement more challenging over time. At the same time, cognitive function plays an equally important role. Executive function, processing speed, and attention are essential for coordinated movement, and research suggests that cognitive decline can often predict mobility deterioration [5].

Psychological factors further influence mobility, particularly the fear of falling. When individuals become overly cautious due to this fear, they may limit their activities, unintentionally accelerating the decline in their mobility. Physical inactivity not only contributes to muscle weakening, but also increases other health risks, further accelerating mobility loss.

In older adults, once mobility limitations occur, reversing them is difficult or even impossible. Therefore, early detection of mobility anomalies is crucial for effective care management and the planning of preventive interventions.

Various instruments have been developed to assess mobility in controlled clinical settings, including the Short Physical Performance Battery (SPPB), Timed Up and Go Test (TUG), Chair Rise Test (CRT), and gait analysis [6,7]. Although these tools provide valuable data, they are typically administered during periodic clinical visits and require specialized equipment and trained personnel. As a result, they may not capture day-to-day fluctuations in mobility or be practical for large-scale, continuous monitoring.

On the other hand, as the global population ages, conventional in-person mobility assessments may become a bottleneck due to limited healthcare resources and the need for specialized personnel. An automated system could ease this strain by enabling remote monitoring and early detection of mobility issues. Whether utilizing wearable sensors, computer vision, or smart home devices, such systems can continuously track mobility in everyday settings. By constantly analyzing movement patterns, they can detect subtle changes or deviations from an individual’s baseline, potentially signaling the onset of a mobility disorder or an increased risk of falls.

### 1.2. Brief Review of the Existing Solutions for Mobility Anomaly Detection

Recent technological advancements opened the way for a range of innovative solutions in the field of automated mobility monitoring. For example, the progress in sensor technology has enabled the creation of compact and accurate wearable devices. These devices can measure a variety of parameters—including acceleration, orientation, step count, blood pressure, and oxygen saturation—and are designed to be seamlessly integrated into everyday clothing or accessories [8,9,10]. In parallel, video cameras along with sophisticated computer vision algorithms are now capable of monitoring gait and other movements [11,12]. When combined with the Internet of Things (IoT), these sensors can transmit real-time data to cloud-based platforms for analysis. Furthermore, artificial intelligence (AI) and machine learning algorithms play a crucial role in processing the vast amounts of data generated [13,14]. These systems can detect patterns and anomalies that may indicate emerging mobility issues, including falls [15,16], and over time, machine learning models become increasingly adept at recognizing subtle deviations from an individual’s typical movement patterns [17,18,19].

Starting from the general definition of anomalies as “patterns in data that do not conform to an expected or well-defined notion of normal behavior” [20], it is important to recognize that detecting such deviations in smart homes depends on the data collected from various sensors. Consequently, existing solutions for mobility anomaly detection can be logically categorized by the type of sensors used—namely, wearable, ambient, and video sensors—each offering a distinct perspective on resident mobility and behavior.

Alternatively, existing solutions can be classified according to the specific facet of mobility anomaly they target. For instance, while many studies focus on fall detection, others are designed to identify prolonged immobility or detect changes in gait. Based on a brief, nonsystematic review of the literature, Table 1 summarizes some of the most frequently addressed mobility anomalies, the sensor types employed, and the corresponding references.

Data in Table 1 make it evident that no single sensor can capture every facet of mobility anomaly. Each sensor type has its strengths and limitations, often stemming from the nature of the data it produces. For instance, video sensors (e.g., RGB or depth cameras) generally provide highly detailed information about a person’s posture, movements, and even facial expressions, making them valuable for tasks like fine-grained gait analysis or precise fall detection. However, these sensors are also intrusive, raising privacy and ethical concerns, and often require significant computational resources to process video streams in real time.

Likewise, wearable sensors such as accelerometers and gyroscopes capture continuous, individualized data on movement dynamics, but they depend on user compliance (e.g., remembering to wear the device) and can sometimes be uncomfortable or inconvenient for older adults. Even among these wearable solutions, battery life, data accuracy, and signal drift can pose additional challenges.

In contrast, binary sensors, like passive infrared sensors (PIR), door contacts, or pressure/force sensors in floors or insoles tend to be far less intrusive, but the data they provide are much coarser; for example, PIR sensors merely detect the presence or absence of motion, while force sensors can register weight shifts without capturing detailed limb movements. This more abstract level of information can suffice for broad anomaly detection (e.g., detecting a lack of movement over time), but it may be insufficient for identifying subtle changes in gait or for distinguishing between a genuine fall and a person sitting abruptly.

Despite these limitations, various solutions relying mainly or exclusively on binary sensors have been described in the literature. For example, in [39], Cejudo et al. present a smart home anomaly detection system that is unique for using data from multiple residences to model behavior at a population level. Each sensor event is mapped to an activity label, so each day per person can be represented as a vector of activity counts/durations. Then, deep learning is applied to learn normal behavior patterns and flag anomalies.

The studies presented in [40,41,42] utilize features such as frequency and duration of visits to specific rooms during defined time intervals, or a measure of time out of home (TOOH) to statistically detect deviations from normal behavior.

In [43], the spatiotemporal characteristics of activities, as reflected by sensor data, are encoded into graphically represented “activity maps,” and anomaly detection is performed through a clustering technique applied to the resulting images.

The solution described in [44] addresses the problem of uncertain sensor data and introduces a probabilistic weighting of inactivity periods, called ‘inactivity score’ IS(t), which reflects the reliability of sensor measurements and does not reset to zero with each detected activity event. Instead, each event probabilistically reduces the current score based on sensor certainty. The inactivity score is calculated recursively and increases with time during periods without reliable activity detection. Higher scores indicate prolonged inactivity or a higher probability of emergency situations.

In [45], behavioral anomalies are identified through Temporal Convolutional Networks (TCNs) that predict future behavioral patterns based on historical sleep profile data. Deviations between predicted and actual behaviors (prediction errors) indicate potential anomalies. The study explicitly addresses mobility through analysis of outings and visits, which are considered indicators of physical and social activity. The ability to perform outdoor activities (such as grocery shopping or social visits) serves as an indirect indicator of mobility and independence.

The method described in [46] relies on a self-organizing learning model to capture the resident’s Circadian Activity Rhythm (CAR)—essentially, the 24 h pattern of movement between different rooms. The system first learns a nominal profile of the resident’s daily sensor usage cycle, effectively modeling what a “normal day” looks like in terms of sensor events at various times. The anomaly detection then works by comparing each day’s sensor pattern to the learned nominal pattern and quantifying deviations.

From the above presentation, it follows that the choice of solution for mobility anomaly detection is not merely about technical feasibility; it also involves practical, ethical, and user-related considerations.

Pavel et al. in [49] identified the following essential constraints and design principles for such systems:

- Economic Feasibility: The system should be affordable for at-risk individuals (e.g., seniors and their families) or covered by the healthcare financing system to ensure accessibility.
- Scalability: It should be cost-effective and capable of being deployed widely to benefit large populations.
- Unobtrusiveness: The monitoring should be as transparent as possible to the individual. Ideally, no wearable or carried devices should be required, relying instead on passive sensors or cameras.
- Continuity of Sensing: The system should collect data frequently or continuously to track patient-specific trends and enable just-in-time interventions.
- Usability: The system should be easy to install, operate, and maintain, requiring minimal effort. It should have features like portability, long battery life, and self-calibration.
- Adaptability: The system must adjust to individual users, various locations, and changing environmental conditions.
- Self-Checking: It should have the capability to monitor and assess its own performance for reliability.
- High Sensitivity and Specificity (Low False Alarms): Accuracy is critical to avoid excessive false alarms while maintaining effective monitoring and event detection.
- Privacy and Security: The system must ensure the security and privacy of both individuals and care teams. This includes authentication, data sharing policies, and protection against unauthorized access.
- Workflow Integration: The system must seamlessly integrate into provider and caregiver workflows without creating additional burdens.

Regarding the actual methods for anomaly detection, a variety of taxonomies have been proposed (see [20,50]), but regardless of different sensor-based and anomaly-specific classifications, it is useful to look at how anomaly detection itself is conceptually structured. Anomaly detection methods generally follow three main steps:

- Defining the norm (i.e., characterizing what “normal” mobility looks like),
- Estimating deviations from that norm (using measures such as distances, probabilities, or reconstruction errors),
- Determining a decision threshold (fixed or dynamically adapted) that marks the boundary between normal and abnormal behavior.

**Figure 1:**
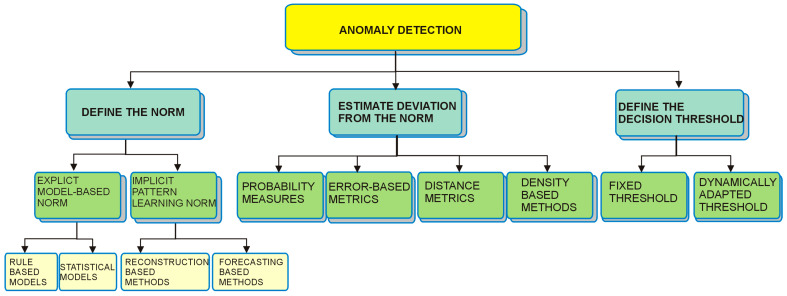
A framework for analyzing the anomaly detection methods.

In Figure 1, we synthesized the methods commonly used in each of these steps.

### 1.3. Objective of This Study

While binary sensors inherently lack the fine-grained detail necessary to capture subtle nuances in gait and balance, their low cost, unobtrusiveness, and ease of deployment for continuous, long-term monitoring present a compelling case for their use. Empirical evidence suggests that these sensors can reliably detect critical mobility anomalies, such as prolonged periods of inactivity, that may indicate falls or rapid declines in mobility, as well as sudden shifts in the living space occupancy that disrupt established activity patterns.

In this context, the objective of the present study is to explore the feasibility, usability, and adaptability of a solution for long-term monitoring and detection of mobility loss based exclusively on non-intrusive, low-cost, binary sensors, compatible with nearly any living space and offering maximum usability and adaptability.

## 2. Method and Datasets

### 2.1. Assumptions

The first assumption underpinning this study is that human mobility is, to a certain extent, predictable. This premise is supported by the seminal work [51], which demonstrated that the theoretical upper limit of predictability for outdoor human mobility is as high as 93%, and by the findings reported in [52], who predicted the next sensor event in the context of indoor mobility tracking with an accuracy of 79–82% in single-resident households.

It is likely that prediction accuracy in real-life applications is lower than the figures cited above, owing to individual variability, sensor noise, and inherent randomness. Nonetheless, human daily activities exhibit significant regularity, which can be learned and predicted, and can serve as a baseline for anomaly detection algorithms.

The second assumption underlying this study is that even a gradual decline in mobility can be detected by monitoring daily activity levels. Studies using in-home sensor networks have demonstrated that reduced daily movements—such as fewer room transitions and longer periods of inactivity—often correlate with declines in clinical measures of mobility. For example, the authors of [53] monitored seniors’ activity over extended periods and found that decreases in sensor-recorded activity were associated with lower scores on standardized assessments of instrumental activities of daily living (IADL), a proxy for functional mobility.

### 2.2. Datasets and Preprocessing

As discussed in [54], the vast majority of available datasets for human activity recognition and anomaly detection are generated by monitoring healthy individuals, thus inherently lacking anomalies. On the other hand, anomalies are, by definition, rare and unpredictable events, meaning that even in cases where datasets do contain anomalies, they are insufficient for training robust machine learning algorithms. Consequently, many studies focusing on anomaly detection in human activity rely on the use of synthetic anomalies generated by manipulating sensor data. This approach, however, raises the question of how well these artificial anomalies emulate natural anomalies and whether synthetic anomalies cover the full spectrum of possible irregularities.

Nevertheless, in the specific case of mobility anomalies, if the datasets used are annotated, it becomes possible to accurately identify intervals characterized by reduced activity—corresponding either to temporary absences from home (outings) or to relaxation periods explicitly marked in the annotations.

Building on this premise, to assess the capability of the solutions proposed in this study to detect point anomalies, we selected two publicly available datasets—specifically, the Aruba and HH120 testbeds from the CASAS dataset [55]. Both testbeds use binary sensors for monitoring the activity of a single resident and feature partial annotations, which allow for precise identification of outings and relaxation periods.

Aruba dataset contains data describing the activities of an elderly woman recorded over a period of 220 consecutive days. Another reason for choosing this dataset is its relative popularity: Alaghbari et al. in [56] identified seven other studies on activity recognition and anomaly detection that use this dataset. HH120 is much shorter (only 64 days) and was included to test the portability of the solution.

The raw data are provided as a text file, with each line representing a sensor event structured as follows:

For the Aruba testbed: Timestamp, Sensor_ID, Sensor_Event, Activity

For the HH120 testbed: Timestamp, Sensor_ID, Location, Sensor_Event, Activity

Data from temperature and light sensors, not directly related to the mobility of the resident, were filtered out from the raw dataset during the preprocessing phase. Additionally, at this stage, we filtered out all OFF-type events recorded by the motion sensors and all CLOSE-type events recorded by the door contact sensors. To mitigate sensor noise, if two successive events recorded by a given sensor were separated by less than one minute, we eliminated the second event in the sequence. Finally, we considered one-hour time intervals and counted the events recorded by each sensor within each interval. This resulted in a CSV file with the following structure:

Date, Hour, Events_count_M001, Events_count_M002, …, Events_count_D003

### 2.3. An Abstraction of the Residential Space

In our previous works [43,57], we argued that, when describing residential living space, a distinction should be made between ‘locations’ and ‘places’, where places are defined as locations imbued with meaning by users, according to their needs, preferences, habits, or values. Rather than being mere physical coordinates, places are shaped by human activity and the intentional decisions behind why people occupy them and for how long. Building on this idea, residential living spaces can be abstracted as a collection of behaviorally meaningful places (BMPs)—such as the living room, bedroom, kitchen, bathroom, and circulation areas—each reflecting the user’s daily activities and routines. By omitting details like the particular layout of the living space, the arrangement of furniture and appliances, and the precise positioning of sensors, this model establishes a framework for monitoring the activities of daily life (ADLs) in nearly any residential setting.

**Figure 2:**
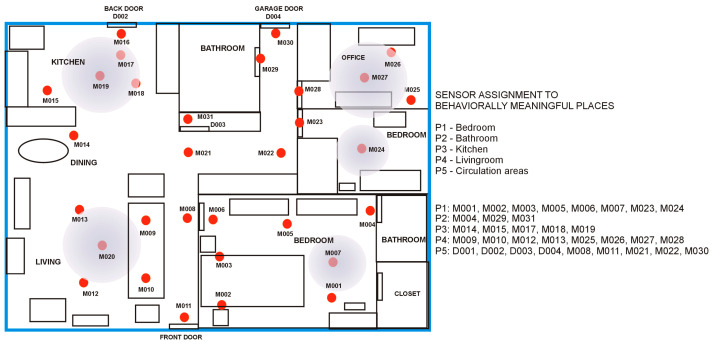
Floorplan with sensor layout and the assignment of sensors to BMPs in Aruba testbed from the CASAS dataset. Mxxx are PIR motion detectors and Dxxx are magnetic door contacts.

With this abstraction, the set of sensors associated with a specific BMP can be treated as a single compound sensor reporting all the events recorded in the respective place. For example, for the sensor layout in the Aruba house from the CASAS dataset, we defined five BMPs and assigned the sensors as shown in Figure 2.

If {P_i_} is the set of sensor events recorded in the place P_i_, and {S_j_} is the set of events generated by sensor S_j_, then{P_1_} = {M001} ∪ {M002} ∪ {M003} ∪ {M005} ∪ {M006 ∪ {M007} ∪ {M023} ∪ {M024} {P_2_} = {M004} ∪ {M029} ∪ {M031} {P_3_} = {M014} ∪ {M015} ∪ {M017} ∪ {M018} ∪ {M019} {P_4_} = {M009} ∪ {M010} ∪ {M012} ∪ {M013} ∪ {M025} ∪ {M026} ∪ {M027} ∪ {M028} {P_5_} = {D001} ∪ {D002} ∪ {D003} ∪ {D004} ∪ {M008} ∪ {M011} ∪ {M021} ∪ {M022} ∪ {M030}                             (1)

Similarly, for the HH120 testbed the sensor-to_BMP assignemnt is{P_1_} = {M009} ∪ {M010} ∪ {M011} ∪ {MA016} {P_2_} = {M008} ∪ {M018} {P_3_} = {MA017} {P_4_} = {M003} ∪ {M004} ∪ {M005} ∪ {M006} ∪ {M007} ∪ {M026} ∪ {MA015} {P_5_} = {D002} ∪ {D004} ∪ {M001} ∪ {M002} ∪ {M011}                                    (2)

### 2.4. Encoding the Activity Starting from Sensor Data

Considering that all sensors have binary output, if $\left|\left\{P_{i}\right\}\right|$ is the cardinal of the set of events {Pi} recorded in the place P_i_ within a specified time slice t_k_, then the overall activity in the entire space is described by a vector in a 5-dimensional space

(3) $$A(t_{k})=[\left|\left\{P_{1}\right\}\right|,\left|\left\{P_{2}\right\}\right|,\;\left|\left\{P_{3}\right\}\right|,\;\left|\left\{P_{4}\right\}\right|,\;\left|\left\{P_{5}\right\}\right|]$$

and the intensity level of the activity (AL) can be estimated by the L2 norm of this vector:

(4) $$AL=\sqrt{\sum_{i=1}^{5}{\left|{\{P}_{i}\}\right|}^{2}}$$

For simplicity, we defined the duration of the time slices, t_k_, as one hour. The successive values of A(t_k_) constitute a time series that synthesizes the spatiotemporal distribution of the monitored individual’s activity over the entire observation period.

### 2.5. Detecting Mobility Anomalies

**Figure 3:**
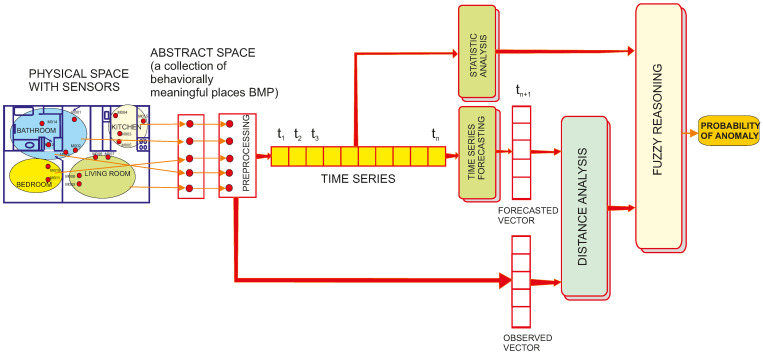
Data processing flow for detecting mobility loss according to the proposed method.

The whole data processing flow for detecting mobility loss is shown in Figure 3.

#### 2.5.1. Selecting the Forecasting Model

Having the data on the spatiotemporal activity of the monitored person structured as a time series A(t_1_), A(t_2_), … A(t_n_), it is possible to forecast values of the series for the next time slice PA(t_n+1_).

Time-series forecasting is a well-established research field with many solutions reporting remarkable forecasting accuracy [58,59]. However, the precision of the predictions often comes at a cost in terms of computational load, an increased risk of overfitting, and requires large training datasets. Therefore, in order to identify a lightweight forecasting model aligned with the usability and adaptability requirements stated above and suitable for the selected datasets, we evaluated several models recognized in existing studies as effective for relatively short time series: Seasonal Autoregressive Integrated Moving Average (SARIMA), Support Vector Regression (SVR), Vector Autoregression (VAR), Random Forest (RF), and Prophet. For this purpose, we computed the Root Mean Squared Error (RMSE) of the forecasts across both test datasets and selected the model with the lowest RMSE. An additional selection criterion was derived from the requirement of maximum adaptability of the solution: the models requiring less tuning when switching between datasets were preferred.

#### 2.5.2. Computing the Probability of Anomaly

By comparing the forecasted values of the activity with the observed data for the respective time interval OA(t_n+1_), for example by means of the Euclidean distance

(5) $$Dist=\sqrt{\sum_{i=1}^{5}{\left({OA}_{i}-{PA}_{i}\right)}^{2}}$$

**Figure 4:**
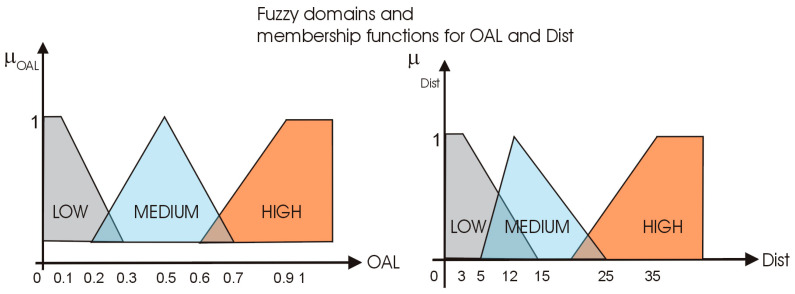
Fuzzy domains and membership functions for OAL and Dist.

We obtain a measure of the deviation of the current distribution of activities across BMPs from the learned activity routine. Since we are only interested in potential mobility loss, i.e., deviations towards lower intensities of activities, we compute the observed activity level OAL as

(6) $$OAL=\frac{AL}{HAL}$$

where AL is calculated with (3) and HAL is the average of the values of AL for the corresponding hour of the day across the entire training dataset. Using OAL and Dist as input variables, we implemented a simple fuzzy reasoning module with three fuzzy domains and linear membership functions, as shown in Figure 4.

The output of these modules is the probability of a mobility anomaly (PA), estimated according to the rule base shown in Table 2.

If PA is higher than a tunable threshold, an alert flag is set.

Algorithm 1 listed below synthesizes the entire data processing workflow for detecting sudden mobility loss.
**Algorithm 1.** Data processing workflow for detecting point mobility anomaliesBEGIN // === **Step 1: Data Preprocessing** === // Load raw sensor data (each record: Timestamp, Sensor_ID, Sensor_Event, etc.) raw_data ← LOAD_SENSOR_DATA(“raw_sensor_file.txt”) // Filter out sensors not related to mobility (e.g., temperature, light) filtered_data ← FILTER(raw_data, sensor_type NOT IN {“temperature”, “light”}) // Remove unwanted events: // - Exclude OFF events for motion sensors. // - Exclude CLOSE events for door sensors. filtered_data ← FILTER(filtered_data,             (sensor_category = “motion” AND event_type ≠ “OFF”) AND             (sensor_category = “door” AND event_type ≠ “CLOSE”)) // Mitigate sensor noise: // For each sensor, if two successive events occur within 60 s, remove the second. debounced_data ← DEBOUNCE(filtered_data, time_threshold = 60 s) // Aggregate events: // Count the number of events per sensor in one-hour intervals. hourly_counts ← AGGREGATE(debounced_data, interval = “1 h”) // === **Step 2: Abstract Residential Space into Behaviorally Meaningful Places (BMPs)** === // Define sensor-to-BMP mappings: BMP1_sensors ← {M001, M002, M003, M005, M006, M007, M023, M024} BMP2_sensors ← {M004, M029, M031} BMP3_sensors ← {M014, M015, M017, M018, M019} BMP4_sensors ← {M009, M010, M012, M013, M025, M026, M027, M028} BMP5_sensors ← {D001, D002, D003, D004, M008, M011, M021, M022, M030} // For each time interval, sum events per BMP. FOR each time_interval IN hourly_counts DO   P1 ← SUM_EVENTS(hourly_counts[time_interval], sensors IN BMP1_sensors)   P2 ← SUM_EVENTS(hourly_counts[time_interval], sensors IN BMP2_sensors)   P3 ← SUM_EVENTS(hourly_counts[time_interval], sensors IN BMP3_sensors)   P4 ← SUM_EVENTS(hourly_counts[time_interval], sensors IN BMP4_sensors)   P5 ← SUM_EVENTS(hourly_counts[time_interval], sensors IN BMP5_sensors)       // Construct the activity vector A(tk) for this interval   activity_vector[time_interval] ← [P1, P2, P3, P4, P5] END FOR // === **Step 3: Encode Activity Level** === // Compute the overall activity level (AL) as the L2 norm of the activity vector. FOR each time_interval IN activity_vector DO   AL[time_interval] ← SQRT( (P1)^2 + (P2)^2 + (P3)^2 + (P4)^2 + (P5)^2 ) END FOR // === **Step 4: Predict and Compare Activity for Mobility Loss Detection** === // Assume a predictive model is available, trained on historical activity data. FOR each new time interval t DO   // Predict the activity vector for the next interval   predicted_vector ← PREDICT_ACTIVITY(activity_vector, current_time = t)      // Retrieve the observed activity vector for the next interval   observed_vector ← activity_vector[t + 1]      // Compute Euclidean distance (Dist) between predicted and observed vectors   Dist ← SQRT( SUM( (observed_vector[i] - predicted_vector[i])^2 for i = 1 to 5 ) )      // Compute Observed Activity Level ratio (OAL)   // HAL is the historical average AL for the corresponding hour (from training data)   HAL ← HISTORICAL_AVERAGE_AL(hour = t + 1)   OAL ← AL[t + 1]/HAL      // === **Step 5: Fuzzy Inference for Mobility Anomaly** ===   // Use OAL and Dist as input to a fuzzy reasoning module.   // The module applies linear membership functions (LOW, MEDIUM, HIGH) and a rule base.   PA ← FUZZY_INFERENCE_MODULE(OAL, Dist)      // Output the estimated probability of mobility anomaly for this interval   OUTPUT(time = t + 1, anomaly_probability = PA)   IF (PA>ALERT_THRESHOLD) THEN ALERT_FLAG=TRUE END FOREND

To detect gradual mobility loss, we employed a statistical approach: we first computed the daily average of the values of AL given by (4), then performed a linear regression on these averaged values over a 21-day period, and analyzed the slope of the regression line.

## 3. Results

### 3.1. Selecting the Forecasting Model

The values of RMSE for the models considered in the evaluation are shown in Table 3. Considering these results and the requirement for minimal tuning when switching between datasets, we selected Prophet as the forecasting model.

**Figure 5:**
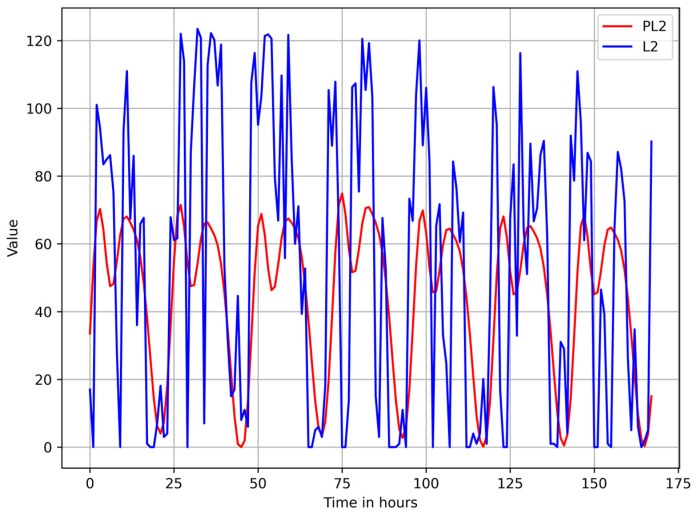
Plot of the observed values of L2 (Euclidean norm of the activity vector) versus PL2 (Euclidean norm of the vector containing the forecasted components of the activity vector) for the Aruba dataset.

**Figure 6:**
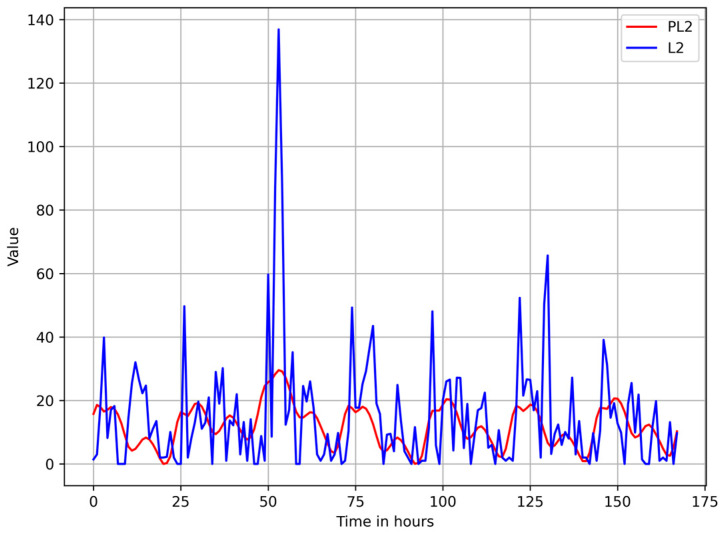
Plot of the observed values of L2 (Euclidean norm of the activity vector) versus PL2 (Euclidean norm of the vector containing the forecasted components of the activity vector) for the HH120 dataset.

Figure 5 and Figure 6 show a plot of the Euclidean norm of the observed activity vector (L2) versus the Euclidean norm of the vector containing the components of the activity vector forecasted with Prophet (PL2) for Aruba and HH120 datasets.

### 3.2. Detecting Point Mobility Anomalies

To evaluate the capability of our proposed method to detect periods of prolonged inactivity as potential mobility anomalies, we examined the Aruba test dataset over a period of 21 days, identifying 60 instances of inactivity lasting longer than one hour. Similarly, within the HH120 dataset, during an identical timeframe of 21 days, we found 36 periods of inactivity exceeding one hour. Nighttime sleep intervals were excluded from the analysis. Then, we compared the known inactivity periods with the instances when the probability of anomaly PA reported by the system was higher than a fixed threshold (0.7).

The results of this evaluation are presented in Table 4, where TP (True Positives) is the number of actual anomalies correctly detected, FN (False Negatives) is the number of actual anomalies that were missed by the detector, FP (False Positives) is the number of normal instances incorrectly flagged as anomalies, and TN (True Negatives) is the number of normal instances correctly identified as non-anomalous. With these values, we computed the following:

(7) $$Detection\;Rate=\frac{TP}{TP+FN}$$

(8) $$False\;Positive\;Rate=\frac{FP}{FP+TN}$$

### 3.3. Detecting Gradual Decline in Mobility

**Figure 7:**
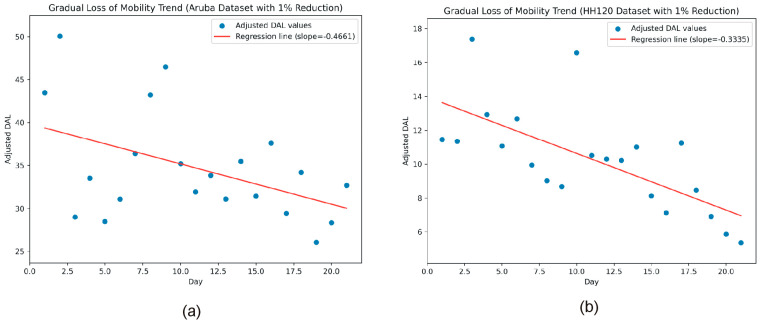
Linear regression of the data on the daily activity levels for the past 21 days with a simulated mobility loss of 1% per day (**a**) with Aruba dataset and (**b**) with HH1210 dataset.

To assess the capability of the described system to detect the gradual loss of mobility, we calculated the daily mean of hourly activity intensity values (AL) using Equation (4) over a period of 21 days. Subsequently, we applied a daily percentage reduction of 1% to the Daily Activity Level (DAL) values. A scatter plot of DAL values across the 21-day interval was generated, followed by performing linear regression analysis on the plotted points. The slope of the regression line was then highlighted to illustrate the mobility trend (see Figure 7).

Besides the slope of the trend line, the system displays additional regression parameters, such as the coefficient of determination *R*^2^, and the *p*-value that provide insight into the magnitude and the statistical significance of the observed mobility decline.

## 4. Discussion

The aforementioned results are promising in what concerns the feasibility of a system capable of detecting both point anomalies and gradual declines in mobility using non-intrusive binary sensors; however, we must acknowledge that the current study is intended only as a proof-of-concept.

In real-life scenarios, it is straightforward to distinguish between outings and mobility anomalies, but it is significantly more challenging to differentiate between normal periods of reduced mobility (e.g., relaxing, reading, watching TV) and situations that are genuinely abnormal in terms of mobility. Also, the simulated gradual mobility decline (1% daily reduction) is an artificial scenario; natural decline may follow nonlinear trends.

Another limitation of the proposed solution is the inherent one-hour latency in anomaly detection, as the comparison between the expected/predicted activity level and the actual observed value is made retrospectively at the end of the time windows. This latency could be significant in emergency situations, such as falls. However, the fact that the system reports an anomaly probability rather than merely flagging moments when this probability exceeds a preset threshold gives it a certain predictive value: by examining the trend of PA values, it is possible, in principle, to anticipate a future degradation in mobility.

The tunable threshold for PA significantly influences detection performance. For example, lowering the anomaly probability threshold from 0.7 to 0.65 increased the detection rate to 92% for the Aruba dataset, albeit with a concurrent rise in the false positive rate to 20%. Proper tuning, tailored to the specific routine of the monitored individual and the sensor distribution, is needed to ensure an optimal trade-off between detection sensitivity and false positive rate.

The performance of the proposed system in terms of anomaly detection accuracy is modest compared to other studies. For instance, the solution described in [60], which uses the same dataset (Aruba), is capable of detecting anomalies in the duration of activities with a sensitivity exceeding 90% for nine out of eleven activities examined. Although it does not directly address the issue of mobility anomalies, study [61] detects synthetic anomalies introduced in the Aruba dataset with a sensitivity of up to 100%.

Future research could bring considerable improvements to our solution in the following directions:

- The number and placement of sensors have a major influence on system performance. Interestingly, the presence of additional sensors in the Aruba testbed did not necessarily lead to better detection outcomes. In fact, when sensors within a single BMP reported a high number of events per hour, challenging spikes were created that proved difficult to predict, thereby reducing the overall predictive accuracy as measured by RMSE. Additional filtering methods during the preprocessing phase could mitigate these spikes, reduce sensor noise, and consequently improve predictive accuracy. Nevertheless, a minimal redundancy in sensor count is beneficial, as it ensures remarkable robustness against sensor faults. During testing, we removed one sensor from each BMP without noticeably affecting anomaly detection accuracy.
- Prediction accuracy is critical for overall system performance; thus, the selection of the Prophet model is not definitive. The Prophet model does demonstrate strong performance in capturing daily and weekly seasonality and robustness in handling outliers. However, alternative predictive models capable of managing multivariate inputs might yield even better results.
- The current configuration of the fuzzy inference system is the simplest possible, comprising only three fuzzy domains with linear membership functions and minimal tuning. Introducing additional fuzzy domains and personalized tuning for specific individuals is likely to yield enhanced anomaly detection outcomes.
- The selection of a one-hour time interval for constructing time series from sensor events is arbitrary and directly influences the system’s latency. Future research should examine the impact of opting for shorter time intervals. Alternatively, the possibility of defining variable-length “behaviorally significant time intervals” (e.g., sleep time, morning routine, peak activity period, daytime rest, etc.) could be investigated.

Despite its limitations, the proposed system provides a promising framework for monitoring and detection of mobility loss in older adults, effectively leveraging several key advantages inherent to the method employed. The system primarily uses binary sensors, which offer substantial benefits including low cost, portability, easy installation, and non-intrusiveness, thereby facilitating wide adoption and continuous long-term monitoring without significantly compromising sensitivity.

Additionally, the forecasting model utilized, Prophet, is computationally lightweight, robust with relatively small datasets, and proficiently captures complex patterns, such as daily and weekly seasonality inherent in human activity. Furthermore, the modular architecture, integrating a fuzzy inference mechanism, allows for easy adjustments and personalization according to the monitored individual’s routine, enhancing adaptability and specificity.

Another notable advantage is the system’s capability to report a probability of anomaly rather than merely triggering alerts when thresholds are exceeded; this provides useful predictive insights, as analyzing trends in anomaly probabilities can potentially enable early anticipation of gradual mobility deterioration.

This framework appears to be a promising research direction towards an economically feasible, scalable, and user-friendly solution for early detection of mobility anomalies.

## 5. Conclusions

This study proposes a proof-of-concept approach exploring the feasibility of employing non-intrusive, low-cost binary sensors, combined with lightweight forecasting models and fuzzy inference, for monitoring and detecting mobility anomalies in older adults. Although initial results indicate that the system can effectively identify periods of prolonged inactivity, suggesting potential falls or mobility issues, the detection accuracy is modest compared to other, more complex or invasive methods.

It is important to acknowledge that the current findings represent only an early demonstration of concept viability. The system shows promise, particularly in addressing some limitations associated with clinical assessments, such as intrusiveness, cost, and practicality for continuous monitoring. Nonetheless, significant future work is essential to refine the solution, enhance detection specificity, and improve the accuracy of predictive modeling.

Future work should focus on refining the anomaly detection algorithms to enhance specificity, possibly by integrating contextual data, adopting more sophisticated fuzzy inference mechanisms, or exploring alternative forecasting models that leverage multivariate inputs. Additionally, real-world deployment trials involving diverse older populations are necessary to validate the system’s effectiveness and user acceptability in everyday living environments. The potential integration of complementary sensor modalities, such as wearables for occasional calibration or advanced deep learning methods for improved forecasting accuracy, should also be considered.

## Figures and Tables

**Table 1:** Summary of the most frequent mobility anomalies addressed in the literature.

Mobility Anomaly	Sensors Used	References
Falls	Depth sensors	[21,22]
	Accelerometers, gyroscopes	[23]
	Passive Infrared (PIR) and accelerometer	[24,25]
	Video	[26]
Gait anomalies	RGB camera	[27]
	Pressure sensors in smart insoles	[28]
	Pressure/force sensors in smart floors	[29,30]
Wandering and erratic walking in dementia	Active infrared sensors	[31]
	Ambient beacons and wearable transponder	[32]
	GPS	[33,34]
Prolonged inactivity	Smartphone sensors	[35,36]
	Microphone + accelerometer	[37]
	Passive infrared (PIR) sensors	[38,39,40,41]
Slow loss of mobility due to chronic conditions	Passive infrared (PIR) sensors	[42,43,44,45,46]
	Electric power load	[47]
Sarcopenia-related mobility anomalies	Smartwatch sensors	[48]

**Table 2:** The rule base of the fuzzy inference module used to estimate the probability of mobility anomaly.

Observed Activity Level (OAL)	Euclidean Distance Between the Observed and Predicted Vectors (Dist)	Probability of Mobility Anomaly (PA)
Low	Low	Medium
Low	Medium	Medium
Low	High	High
Medium	Low	Low
Medium	Medium	Medium
Medium	High	Medium
High	Low	Low
High	Medium	Low
High	High	Low

**Table 3:** RMSE values for the forecasting models tested.

Testbed	Model	Short Length Train Dataset (28 Days)	Full Length Train Dataset
Aruba	Prophet	23.67	19.47
	RF	19.03	19.83
	SVR	20.78	20.16
	SARIMA	21.36	18.39
	VAR	21.28	21.46
HH120	Prophet	7.95	7.13
	RF	7.44	7.33
	SVR	8.03	7.5
	SARIMA	8.9	7.73
	VAR	8.53	8.14

**Table 4:** Evaluation metrics for point mobility anomaly detection.

Dataset	TP	FN	FP	TN	Detection Rate	False Positive Rate
Aruba	49	11	56	281	0.81	0.16
HH120	28	8	42	295	0.77	0.12

## Data Availability

The data presented in this study were derived from the following resources available in the public domain: CASAS Activity Recognition Datasets. Available online: https://data.casas.wsu.edu/download/ (accessed on 8 January 2025). The source code of the main modules is available at: https://github.com/isusnea/anomaly_detection (accessed on 20 April 2025).
